# Artificial intelligence in global health: An unfair future for health in Sub-Saharan Africa?

**DOI:** 10.1093/haschl/qxaf023

**Published:** 2025-02-05

**Authors:** Audêncio Victor

**Affiliations:** Public Health Postgraduate Program, School of Public Health, University of São Paulo, São Paulo, SP 01246-904, Brazil; Department of Nutrition, Ministry of Health, Zambezia 2400, Mozambique

**Keywords:** artificial intelligence, health inequalities, Africa, social justice in health, global governance

## Abstract

Artificial intelligence (AI) holds transformative potential for global health, particularly in underdeveloped regions like Africa. However, the integration of AI into healthcare systems raises significant concerns regarding equity and fairness. This debate paper explores the challenges and risks associated with implementing AI in healthcare in Africa, focusing on the lack of infrastructure, data quality issues, and inadequate governance frameworks. It also explores the geopolitical and economic dynamics that exacerbate these disparities, including the impact of global competition and weakened international institutions. While highlighting the risks, the paper acknowledges the potential benefits of AI, including improved healthcare access, standardization of care, and enhanced health communication. To ensure equitable outcomes, it advocates for targeted policy measures, including infrastructure investment, capacity building, regulatory frameworks, and international collaboration. This comprehensive approach is essential to mitigate risks, harness the benefits of AI, and promote social justice in global health.

## Introduction

Artificial intelligence (AI) has emerged as a transformative force in global health, promising significant advancements in diagnostics, treatment personalization, and resource optimization.^1-4^ However, the application of AI in underdeveloped regions such as Sub-Saharan Africa raises critical concerns about the perpetuation and amplification of existing inequalities.^5,6^ Africa, which bears 25% of the global disease burden, has only 3% of the world's healthcare professionals, an alarming disparity that could be exacerbated by the inadequate adoption of AI.^7,8^ The lack of infrastructure and the scarcity of high-quality data are central challenges that hinder the effective implementation of AI in the region, potentially compromising the adaptation of AI algorithms to local realities, resulting in ineffective diagnoses and treatments.^6,9^

Moreover, Africa's limited digital infrastructure, where only 28% of the Sub-Saharan population regularly accesses the internet, exacerbates these challenge.^10^ The risk of unfair results produced by AI systems is also a significant concern. Algorithms trained on predominantly Western data may fail when applied to African populations, leading to inaccurate diagnoses and inadequate treatments.^11^ The absence of rules and policies to guide how AI is used in many African countries exacerbates this risk, allowing AI to be implemented without the necessary ethical and legal safeguards.^12^

The adoption of AI in African healthcare is also deeply influenced by global economic dynamics that favor developed countries and leave African nations dependent on external technologies.^13,14^ This technological and financial dependency not only perpetuates global inequalities but also limits African countries’ sovereignty in defining their own health strategies.^15^ Thus, the implementation of AI in Africa must be carefully considered to avoid exacerbating health inequalities, promoting instead an approach that prioritizes social justice and inclusion.

## Challenges in AI implementation in Africa

### Lack of structured data and inadequate infrastructure

One of the greatest challenges for effective AI implementation in Africa is the lack of systematic and well-structured data. Artificial intelligence algorithms rely on large volumes of high-quality data to train accurate and reliable models.^16^ However, most health data available globally comes from developed countries, with only 1% of data originating from African countries.^9^ This lack of representation in the data can lead to algorithms that do not reflect local realities and needs, resulting in diagnoses and treatment recommendations that may be inadequate or even harmful.^17-20^ Additionally, Africa's computational infrastructure is insufficient to support the large-scale implementation of AI technologies.^10^ Internet connectivity is a significant obstacle in Sub-Saharan Africa, where only 28% of the population has regular internet access. This limitation compromises the viability of AI based health systems, as most global science and publications come from wealthy countries with minimal representation in global health production. Moreover, most of the global science and scientific publications are produced by wealthy countries, resulting in an insignificant representation of African contribution.^9,21^

### Biases and fairness in AI

Another significant challenge is the risk of unfair results produced by AI systems. Algorithms trained on data from predominantly white populations often fail when applied to African populations, leading to inaccurate diagnoses and inadequate treatments for diseases prevalent in the region.^11^ For example, a study conducted by Bellemo et al.^20^ showed that despite good results in diabetic retinopathy screening in Zambia, the overall effectiveness of AI models can be compromised by biases in the training data. Moreover, the absence of a robust regulatory framework in many African countries exacerbates this risk, allowing AI to be implemented without the necessary ethical and legal safeguards.^12^

An example of this is the consistent underestimation of the severity of health conditions in Black patients, which has been observed in different global contexts.^22,23^ If such algorithms are applied directly in Africa, where the majority of the population is Black, it is likely that these biases will be perpetuated and even amplified, further aggravating health inequalities.^11,24^

### Brain drain and financial investments

The implementation of AI in healthcare also faces the challenge of brain drain. Africa already suffers from a shortage of healthcare and technology professionals, and the adoption of AI may exacerbate this situation by increasing the demand for specialists who often migrate to developed countries in search of better working conditions and remuneration.^7,8^ It is estimated that up to 20 000 healthcare professionals leave the African continent annually in search of better opportunities abroad.^9^ This not only depletes the human resources needed for the effective implementation of AI but also worsens the health crisis in many African countries.

Financially, global investment in AI for health surpassed 11 billion dollars in 2021, with countries like the United States and the United Kingdom leading these investments. In contrast, most African countries invest only a fraction of this amount, often relying on external aid to fund technological initiatives in healthcare.^12^ This not only depletes the human resources needed for the effective implementation of AI but also worsens the health crisis in many African countries.

### Global economic dynamics and the role of capitalism

The adoption of AI in healthcare is also deeply rooted in global economic dynamics. Developed countries invest billions of dollars in the development of AI technologies, consolidating their economic and scientific dominance.^25^ In contrast, African countries, due to their limited infrastructure and restricted economic resources, become dependent on externally developed technologies, often without these technologies being adapted to local needs.^9,12^ This imbalance perpetuates global inequality, leaving African countries at a disadvantage both economically and technologically. Capitalism, with its inherent tendency to generate wealth and power concentration, has historically shown an ability to create deep inequalities both within and between nations. Artificial intelligence, as a product of this system, can easily follow the same path, favoring the economic interests of developed countries and leaving developing nations even more marginalized.^13,14^ Historically, the tendency to prioritize nationalist interests at the expense of the global common good has exacerbated these disparities, as evidenced by the unequal distribution of COVID-19 vaccines.^26^ In Africa, access to vaccines was severely limited, resulting in vaccination rates of only 10% in some regions, while developed countries had already vaccinated most of their populations.^26-28^

### Geopolitical tensions and AI governance

A crucial but often overlooked factor in AI implementation in global health is the role of geopolitical power struggles. The United States past decision to withdraw from the World Health Organization (WHO) during the COVID-19 pandemic, coupled with discussions of a possible future withdrawal under a new administration, has significant implications for global health governance.^29-32^ The WHO plays a fundamental role in setting global health policies, regulating AI in health applications, and ensuring equitable access to new technologies. The potential weakening of the WHO due to reduced financial contributions from major powers like the United States could disproportionately affect low- and middle-income countries, which rely on global governance structures for policy guidance and technological support.^31^ Furthermore, AI development has become a battleground for global superpowers, with the United States, China, and the European Union leading investments and setting technological standards.^25,33^ This competition raises concerns about how AI health applications are designed and implemented in regions like Africa, where there is limited agency in shaping these innovations. The dominance of Western and Chinese AI models may lead to systems that fail to consider the specific epidemiological, social, and economic realities of African nations, perpetuating inequities rather than alleviating them.^34-37^

## Future perspectives and ethical considerations

For AI to be truly beneficial for Africa, it is essential to adopt an approach that prioritizes inclusion and equity. This includes building robust infrastructures, collecting locally relevant data, and developing rules and policies to guide how AI is used to ensure fairness and ethical practices. The lack of digital infrastructure and health governance in many African countries limits these nations’ ability to effectively regulate and implement AI technologies. Moreover, it is crucial that African countries actively participate in the development of these technologies, ensuring that their needs and realities are reflected in the proposed solutions.^5,6^

## Policy implications and recommendations

To effectively address these challenges and maximize the benefits of AI, several policy measures are essential. First, governments and stakeholders must prioritize investments in digital and computational infrastructure to create a robust foundation for AI applications in healthcare.^16^ Simultaneously, ethical and legal regulatory frameworks should be established to promote fairness, accountability, and transparency in AI deployment.^12^ Local capacity building also plays a crucial role, requiring the development of partnerships aimed at training African data scientists and healthcare professionals to reduce dependency on external expertise.^24^ Finally, fostering international collaboration is key; initiatives that unite governments, academia, and the private sector can drive the development of AI technologies specifically tailored to address the unique needs and realities of African contexts.^38^

## Conclusion

AI has the potential to transform healthcare in Africa, but its impact will strongly depend on how these technologies are implemented and regulated. Without careful consideration of local contexts and global dynamics, there is a risk that AI could exacerbate rather than mitigate health inequalities. Additionally, the shifting geopolitical landscape and the role of global institutions must be considered to ensure that AI policies are equitable and that all regions have an active voice in shaping the future of healthcare technologies.

## Supplementary Material

qxaf023_Supplementary_Data
