# Supplementary material for: Artificial intelligence in global health: An unfair future for health in Sub-Saharan Africa?
Source: Health Aff Sch. 2025 Feb 5;3(2):qxaf023. doi: 10.1093/haschl/qxaf023 (PMC11823112; doi:10.1093/haschl/qxaf023)
Supplement: qxaf023_Supplementary_Data [file qxaf023_supplementary_data.zip › Disclousure Form.docx]

**Health Affairs Scholar**
**Author Disclosure Form**

**Manuscript Title:**
"Artificial Intelligence in Global Health: An Unfair Future for Health in Sub-Saharan Africa?"

**Corresponding Author:**
Audêncio Victor, BSc, MPH
School of Public Health
University of São Paulo
Email: audenciovictor@gmail.com

**List of Authors:**

- Audêncio Victor

**1. Conflicts of Interest:**

- **Audêncio Victor**: Declare that there are no financial or personal relationships with other people or organizations that could inappropriately influence (bias) this work.

**2. Sources of Funding:**

- This research received no specific grant from any funding agency in the public, commercial, or not-for-profit sectors.

**3. Author Contributions:**

- **Audêncio Victor**: Conceptualization, Data curation, Formal analysis, Investigation, Methodology, Project administration, Supervision, Validation, Visualization, Writing – original draft, Writing – review & editing.

**4. Acknowledgements:**

- Not applicable.

**5. Permissions:**

- Confirm that all necessary permissions have been obtained for the use of copyrighted material contained within the manuscript.

**6. Ethical Approval:**

- Not applicable. This manuscript does not involve any active research involving human or animal subjects.

**7. Previous Publication/Consideration:**

- This manuscript has not been published elsewhere and is not under consideration by another journal.

**Signature:**

**Date:** 11 December 2024
